# Balancing the Affinity and Tumor Cell Binding of a Two-in-One Antibody Simultaneously Targeting EGFR and PD-L1

**DOI:** 10.3390/antib13020036

**Published:** 2024-05-02

**Authors:** Julia Harwardt, Felix Klaus Geyer, Katrin Schoenfeld, David Baumstark, Vera Molkenthin, Harald Kolmar

**Affiliations:** 1 Institute for Organic Chemistry and Biochemistry, Technical University of Darmstadt, Peter-Grünberg-Strasse 4, 64287 Darmstadt, Germany; 2 2bind GmbH, Im Gewerbepark D19a, 93059 Regensburg, Germany; 3 Centre for Synthetic Biology, Technical University of Darmstadt, 64287 Darmstadt, Germany

**Keywords:** antibody affinity maturation, bispecific antibody, Two-in-One antibody, yeast display, EGFR binding, PD-L1 binding

## Abstract

The optimization of the affinity of monoclonal antibodies is crucial for the development of drug candidates, as it can impact the efficacy of the drug and, thus, the dose and dosing regimen, limit adverse effects, and reduce therapy costs. Here, we present the affinity maturation of an EGFR×PD-L1 Two-in-One antibody for EGFR binding utilizing site-directed mutagenesis and yeast surface display. The isolated antibody variants target EGFR with a 60-fold-improved affinity due to the replacement of a single amino acid in the CDR3 region of the light chain. The binding properties of the Two-in-One variants were confirmed using various methods, including BLI measurements, real-time antigen binding measurements on surfaces with a mixture of both recombinant proteins and cellular binding experiments using flow cytometry as well as real-time interaction cytometry. An AlphaFold-based model predicted that the amino acid exchange of tyrosine to glutamic acid enables the formation of a salt bridge to an arginine at EGFR position 165. This easily adaptable approach provides a strategy for the affinity maturation of bispecific antibodies with respect to the binding of one of the two antigens.

## 1. Introduction

Over the last few decades, monoclonal antibodies (mAbs) have become an important class of therapeutics for the treatment of various diseases. To increase efficacy and reduce side effects, high affinity and specificity of an antibody to its antigen is essential [1]. An antigen-binding paratope consists of six complementarity-determining regions (CDRs), three of the heavy chain and three of the light chain [2]. During antibody affinity maturation, CDRs undergo a high degree of somatic mutation. In mammals, the generation of high-affinity antibodies occurs in activated B cells by the mutagenic diversification of the variable regions of immunoglobulin (Ig) genes, a process called somatic hypermutation (SHM) [3,4]. As a result of an alternating process between the stochastic SHM of Ig genes and the selection and clonal expansion of B cells that contain affinity-enhanced mutations, the affinity of antibodies increases massively during an immune response [5].

Therapeutic antibodies can be derived from a variety of sources, such as mice, rats, rabbits, chickens, or non-human primates [6,7]. The advantage of animal immunization is that the antibodies have already undergone in vivo affinity maturation through several rounds of SHM [8]. However, even an in vivo-derived antibody may not have the desired affinity for the antigen, which, subsequently, can be optimized by in vitro affinity maturation [9]. For this, random or targeted mutagenesis can be applied [8]. Using the random mutagenesis method, a wide range of mutants can be generated from native antibodies by error-prone PCR, with the mutations occurring randomly rather than selectively [8,10]. The targeted mutagenesis method uses site-directed mutagenesis to produce a library of antibody variants, where the mutations can be designed to occur exclusively in the CDR regions [8,11]. The best matured antibody variant can be isolated by an affinity screening process using a display panning technology such as phage display [12], yeast surface display [13], or ribosome display [14,15]. However, for an antibody library including all possible combinations of single and multiple mutations at all CDRs of a usual variable domain, a library size exceeding 10^30^ variants would be required, making it infeasible to screen using a common display technology [8]. Hot-spot mutagenesis [16], look-through mutagenesis [17], and simultaneous mutagenesis [18] are strategies for improving CDR diversification efficiency and reducing library size.

Mutagenesis approaches have been used in several studies to improve the binding affinity of mAbs. Tillotson et al. used the random mutagenesis approach via error-prone PCR followed by yeast surface display screening to isolate single-chain variable fragment (scFv) mutants that target the transferrin receptor (TfR) with a 3-7-fold-improved binding affinity [19]. A panel of Fab antibody fragments with up to a 10-fold improvement in affinity to the tetravalent antigen streptavidin were obtained by Beucken et al. after the selection of a yeast-displayed library diversified by error-prone PCR [20]. Hu et al. utilized the targeted diversification of multiple CDR regions of a humanized anti-receptor tyrosine kinase 2 (ErbB2) antibody together with phage display to identify a mutant with a 158-fold-increased affinity [21].

Optimizing the affinity of monospecific antibodies can impact the efficacy of a drug and, therefore, the dose and dosing regimen, limit adverse effects, and significantly reduce therapy costs, making optimal affinity a critical factor of drug discovery [10,22,23]. In the case of bispecific antibodies (bsAbs), the affinity of the drug needs to be optimized with respect to two targets [24]. BsAbs are remarkable therapeutic entities for cancer treatment as they are able to cross-link receptors or block two disease-related signaling pathways [25,26,27]. By simultaneously targeting two cancer-specific antigens on the same malignant cell, the tumor specificity of bsAbs can be enhanced [28]. Two therapeutic targets that are upregulated in many solid tumors are programmed death-ligand 1 (PD-L1) and epidermal growth factor receptor (EGFR) [29,30]. We recently reported the isolation of a chicken-derived Two-in-One antibody (HCP-LCE) that simultaneously targets PD-L1 and EGFR with two independent paratopes on a single Fab fragment [31]. Two-in-One antibodies are symmetrical molecules consisting of two identical heavy and light chains, meaning that additional engineering of constant chains is not required [32]. This antibody was the first Two-in-One antibody to be isolated by the separate immunization of two chickens with the EGFR and PD-L1 extracellular domains, followed by combining the respective heavy and light chain modules into a Fab format to isolate antibody variants that bind both targets simultaneously. HCP-LCE inhibits EGFR signaling by binding to dimerization domain II and blocks the PD-1/PD-L1 interaction, exhibiting moderate individual affinity for each antigen [31].

In this study, we investigated whether a Two-in-One antibody could have its affinity optimized for the binding of one target molecule (EGFR) without losing the binding of the second functionality (PD-L1). To our knowledge, the affinity maturation of a Two-in-One antibody obtained by animal immunization and combinatorial screening is described for the first time herein. We optimized the affinity of HCP-LCE regarding EGFR binding via site-directed mutagenesis and yeast surface display (YSD) in combination with fluorescence-activated cell sorting (FACS). By randomizing individual amino acids of the light chain CDR1 (LCDR1) and CDR3 (LCDR3), Two-in-One variants that exhibit a 60-fold improvement in EGFR binding affinity due to the replacement of a single amino acid at LCDR3 position three were isolated, while PD-L1 binding was not impaired. AlphaFold-based modeling predicted that the increased affinity is caused by the formation of an additional salt bridge.

## 2. Materials and Methods

### 2.1. Yeast Library Construction and Screening

For yeast library construction, primers degenerated via NNS coding at one of the nine amino acids of LCDR1 or at one of the 12 amino acids of LCDR3 were designed. For each mutated position, two PCR reactions each containing 200 µM dNTPs (New England Biolabs, Ipswich, MA, USA), 0.5 µM forward primer, 0.5 µM reverse primer, 10 ng LCE template DNA, and 10 U Q5^®^ High-Fidelity DNA Polymerase (New England Biolabs) in Q5 Reaction Buffer (New England Biolabs) were performed. The PCR mixture was subjected to an initial denaturation step of 98 °C (30 s) followed by 30 cycles of 98 °C (10 s), 68 °C (20 s), and 72 °C (30 s). This was followed by a final elongation at 72 °C for 300 s. Both PCR fragments were ligated during a second PCR under the same conditions, resulting in a full-length VL sequence. Subsequently, VL genes were transferred into a pYD_1_-derived vector via homologous recombination in yeast (*Saccharomyces cerevisiae* strain BJ5461) as described previously [31]. Library generation in BJ5464 cells was conducted according to Benatuil and colleagues [33]. To combine the light chain diversity with the heavy chain of HCP-LCE for subsequent Fab display, yeast mating was performed as described before [34].

The induction of gene expression and Fab surface presentation was achieved by the inoculation of yeast cells in Synthetic Galactose minimal medium with Casein Amino Acids (SG-CAA) at an OD_600_ of 1.0 and incubation overnight at 30 °C and 180 rpm. For library sorting, the cells were harvested by centrifugation, washed with PBS + 0.1% (*w*/*v*) BSA (PBS-B) and incubated with 500 nM EGFR-Fc (round 1), 100 nM EGFR-His_6_ + 500 nM HCP-LCE (round 2) or 50 nM EGFR-His_6_ (round 3) for 30 min on ice. Following a PBS-B wash, the cells were incubated with a goat anti-human-Lambda AF647-conjugated F(ab’)2 antibody (SouthernBiotech, Birmingham, AL, USA, diluted 1:75) to detect Fab surface presentation and an anti-human IgG Fc PE-conjugated antibody (Thermo Fisher Scientific, Waltham, MA, USA, diluted 1:50) to detect target binding or with a goat anti-human–Lambda PE-conjugated F(ab’)2 antibody (SouthernBiotech, diluted 1:75) and a 6xHis-Tag AF647-conjugated antibody (Thermo Fisher Scientific, diluted 1:75) for 15 min on ice. An additional PBS-B washing step was followed by FACS screening using a Sony SH800S device.

### 2.2. Expression and Purification of Two-in-One Variants

The reformatting, expression, and purification of full-length antibodies was performed as described previously [31,35]. Isolated yeast vectors were sequenced, and the VL fragment was reformatted into pTT5-derived vectors by golden gate assembly. For the soluble expression of full-length chimeric antibodies, Expi293F^TM^ cells (Thermo Fisher Scientific) were transiently transfected using the ExpiFectamine^TM^ 293 Transfection Kit (Thermo Fisher Scientific) following the manufacturer’s protocol. For purification, cell culture supernatants were collected five days post transfection, sterile-filtered, and applied to a MabSelect^TM^ PrismA HP column (GE Healthcare, Piscataway, NJ, USA) using an ÄKTA pure^TM^ chromatography system (GE Healthcare). One-armed molecules were captured by IMAC (HisTrap HP, GE Healthcare), followed by Strep-Tactin XT affinity chromatography according to the manufacturer’s protocol. Buffer exchange against PBS was performed using a HiTrap^TM^ Desalting column (GE Healthcare).

### 2.3. Cell Lines

Expi293F^TM^ cells were cultured in Expi293^TM^ Expression Medium (Thermo Fisher Scientific), sub-cultured every 3–4 days, and incubated at 37 °C and 8% CO_2_. A431 and A549 cells were cultured in Dulbecco’s Eagle Medium (DMEM, Thermo Fisher Scientific) supplemented with 10% FBS (Merck Millipore, Burlington, MA, USA) and 1% Penicillin–Streptomycin (Sigma Aldrich, St. Louis, MI, USA) in T75 cell culture flasks at 37 °C and 5% CO_2_ and sub-cultured every 3–4 days. Jurkat cells were maintained in RPMI-1640 supplemented with 10% FBS and 1% Penicillin–Streptomycin, sub-cultured every 3–4 days, and incubated at 37 °C and 5% CO_2_.

### 2.4. Thermal Shift Assay

Experiments to determine the thermal stability of the antibody variants were performed using a CFX Connect Real-Time PCR Detection System (BioRad, Hercules, CA, USA) with a temperature gradient from 20 °C to 95 °C and 0.5 °C/10 s. The derivatives of the melting curves were calculated using the corresponding BioRad CFX Maestro 3.0 software to determine the melting temperature (T_M_). All reactions were performed in PBS in the presence of 1 mg/mL protein and SYPRO Orange (Thermo Fisher Scientific, diluted 1:100).

### 2.5. YSD-Based Epitope Mapping

EGFR epitope mapping on the subdomain level was performed using yeast cells displaying truncated versions of EGFR-ECD (amino acids 1–124, 1–176, 1–294, 273–621, 294–543, and 475–621), as previously described [36,37]. The cells were harvested by centrifugation, washed once with PBS-B, and incubated with 250 nM of the respective antibody for 30 min on ice. Surface presentation was verified using an FITC conjugated anti-c-myc antibody (Miltenyi Biotech, Bergisch Gladbach, Germany, diluted 1:75). Separately, antibody binding was verified using an anti-human IgG Fc PE-conjugated antibody (Thermo Fisher Scientific, diluted 1:50). The cells were analyzed by flow cytometry using the CytoFLEX S System (Beckman Coulter, Brea, CA, USA).

### 2.6. Biolayer Interferometry

For affinity determination via biolayer interferometric measurements, the Octet RED96 system (ForteBio, Sartorius, Göttingen, Germany) was used. Anti-human IgG-Fc capture (AHC, Sartorius, Göttingen, Germany) biosensors were soaked in PBS pH 7.4 for at least 10 min before the start of the assay and subsequently used for the immobilization of the HCP-LCE variants. A quenching step in kinetics buffer (KB, Sartorius, Göttingen, Germany) was followed by an association step using EGFR-ECD or PD-L1-ECD at concentrations ranging from 250 nM to 7.81 nM or 500 nM to 7.81 nM, respectively, followed by a dissociation step in KB. Data analysis was performed using ForteBio data analysis software 9.0.0.14. Binding kinetics, including the equilibrium constant K_D_, were determined using Savitzky–Golay filtering and a 1:1 Langmuir binding model. 

For the PD-1 competition assay, HCP-LCE and LCE-E were immobilized on anti-human Fab-CH1 2nd-Generation (FAB2G, Sartorius, Göttingen, Germany) biosensors. Subsequently, 250 nM PD-L1-ECD pre-incubated with either 0 nM, 250 nM, or 500 nM PD-1 was applied for 600 s. 

For the simultaneous binding assay, AHC biosensors were loaded with one-armed (oa) versions of HCP-LCE or LCE-E. After the measurement of the association to 250 nM PD-L1 for 300 s, the association to 250 nM EGFR was determined for 300 s. As controls, oaHCP-LCE and oaLCE-E were incubated with PD-L1 only.

### 2.7. switchSENSE^®^ Measurements

switchSENSE^®^ measurements were performed in a helix^+^ instrument (Dynamic Biosensors, Munich, Germany) using standard adapter chips (ADP-48-2-0, Dynamic Biosensors) in the static fluorescence proximity sensing (FPS) mode. In preparation of the measurement, the target proteins were conjugated to the ligand strand using the heliX^®^ Amine Coupling Kit 1 (HK-NHS-1, Dynamic Biosensors) according to the user manual. Protein–DNA conjugates were purified in the proFIRE^®^ (Dynamic Biosensors). Two Protein–DNA conjugates were separated for both proteins. The earlier eluting conjugate (conjugate 1) was used for the experiments. PD-L1-ligand strand conjugates were hybridized with adapter strand 1 with red dye Ra (AS1-Ra, Dynamic Biosensors) and EGFR–ligand strand conjugates were hybridized with adapter strand 1 with green dye Ga (AS1-Ga, Dynamic Biosensors,) for 20 min at 25 °C. For the single-protein surface experiments, 20% PD-L1-LS/AS1-Ra or 20% EGFR-LS/AS1-Ga was mixed with 80% complementary anchor strand 1 (c-Anchor 1, DC-0, Dynamic Biosensors). For the dual-protein surface experiments, the functionalization mix for electrode 1 contained 10% PD-L1-LS/AS1-Ra, 10% EGFR-LS/AS1-Ga, and 80% complementary anchor strand 1 (c-Anchor 1, DC-0, Dynamic Biosensors). The functionalization mix for electrode 2 contained a ligand-free strand (LFS-0, Dynamic Biosensors) hybridized with adapter strand 2 with the respective dyes (AS-2-Ra and AS-2-Ga, Dynamic Biosensors) and mixed with c-Anchor 2 (DC-0, Dynamic Biosensors) in corresponding ratios. The workflow was set up in heliOS software v2024.1.0. The surface was functionalized for 200 s. The analytes were diluted to 1E-8 M, 1E-9 M, and 1E-10 M in 1x PE140, diluted from the 10-fold-concentrated buffer stock (BU-PE-140-10, Dynamic Biosensors), which was used as a running buffer. Association was measured for 180 s with a flow rate of 200 µL/min. Dissociation was measured for 1800 s with a flow rate of 500 µL/min. In the PD-L1-only experiments, the red LED power was set to 2%, and the green LED power was set to 0%. In the EGFR-only experiments, the green LED power was set to 2%, and the red LED power was set to 0%. In the dual-protein/dual-color experiments, both LEDs were used with 2% power. The measurement was performed at 25 °C. The measured binding curves were evaluated with the heliOS software v2024.1.0. The binding curves obtained in the target-containing electrode were referenced against the target-free electrode. A binding curve with 0 nM analyte was subtracted as a blank. The resulting double-referenced data points were fitted by applying the monophasic association–biphasic dissociation model with continuous amplitudes. The resulting fit curves were plotted using the R package ggplot2.

### 2.8. Cellular Binding

The cellular binding of the HCP-LCE variants was determined by affinity titration using EGFR and PD-L1 double-positive A549 cells. The cells (10^5^ cells/well) were washed with PBS-B and subsequently incubated with the corresponding antibody construct in varying concentrations (2.56 pM–200 nM, serial dilution) for 30 min on ice. Followed by another PBS-B washing step, anti-human IgG Fc PE-conjugated antibody (Thermo Fisher Scientific, diluted 1:50) was applied for 20 min on ice. After final washing with PBS-B, flow cytometry was performed using the CytoFLEX S System (Beckman Coulter). The relative fluorescence units (RFUs) were plotted against the respective logarithmic antibody concentration. The resulting curved were fitted with a variable slope four-parameter fit using GraphPad Prism.

### 2.9. Real-Time Interaction Cytometry (RT-IC)

The real-time kinetics on the cells were measured in a heliX^cyto^ device (Dynamic Biosensors) with chip type M5 (CY-M5-1, Dynamic Biosensors). Antibodies were labeled with the red dye using the heliX^cyto^ labeling kit red dye 1 (CY-LK-R1-1, Dynamic Biosensors) according to the instructions in the manual. The degree of labeling (DOL) was determined by photometric measurements. The determined DOLs were 2.5 and 4 for HCP-LCE and LCE-E, respectively. The cells were resuspended in PBS and strained through a 30 µm cell strainer (FIL-30-20, Dynamic Biosensors). Except for one measurement of LCE-E on A431 cells, all interactions were measured with cells that had been treated with 2% PFA for 15 min at room temperature. The cells were used at a concentration of 2 × 10^6^ cells/mL for capturing. The antibodies HCP-LCE and LCE-E were diluted to 60/20/6.67 nM and 100/50/25 nM, respectively. We used 1xPPBS, diluted from a 10-fold stock (BU-RB-10-1, Dynamic Biosensors), for antibody dilution and as a running buffer. The workflow was set up in the heliOS software v2024.1.0. Signals were normalized with normalization solution for the red dyes (NOR-0, Dynamic Biosensors) at the respective concentration. Red LED power was set to 0.11–0.13%. The measurement was carried out at 25 °C, and the autosampler was cooled to 4 °C. Each association was measured for 300 s, and dissociation was measured for 1800 s. The measured binding curves were evaluated using heliOS software v2024.1.0. The binding curves in electrode 1 and electrode 2 were normalized according to the signal obtained with the normalization solution. The signals obtained in the cell trap-containing electrode were referenced against the trap-free electrode. The resulting referenced data points were fitted by applying the monophasic association–biphasic dissociation model with discontinuous amplitudes and with the end of dissociation set to zero. The data points, fit curves, and calculated parameters of the fit model were plotted using the R package ggplot2. Half-lives were approximated by applying the Newton–Raphson method provided in the R package animation.

### 2.10. AlphaFold-Based Modeling

AlphaFold2 modeling was performed with ColabFold version 1.5.5 [38,39,40,41]. Multiple sequence alignments (MSAs) were generated by MMseq2 [42,43] using the databases of UniRef100 [44]. The final model was relaxed by the AMBER force field [45]. Binding energies were calculated using PRODIGY [46,47], and the dissociation constant calculated for a temperature of 25 °C. Therefore, the top-ranked structure of the trimeric VH:VL and antigen domain complex was selected based on residue confidence scores (pIDDT) and error plots (PAE). The tetrameric complex was generated by modeling VH:VL with EGFR (P00533; L25-S645) using AlphaFold2 and the subsequent docking of PD-L1 (Q9NZQ7; N17-P227) utilizing HDOCK [48,49].

## 3. Results

### 3.1. Yeast Surface Display Library Generation and Screening

**Figure 1:**
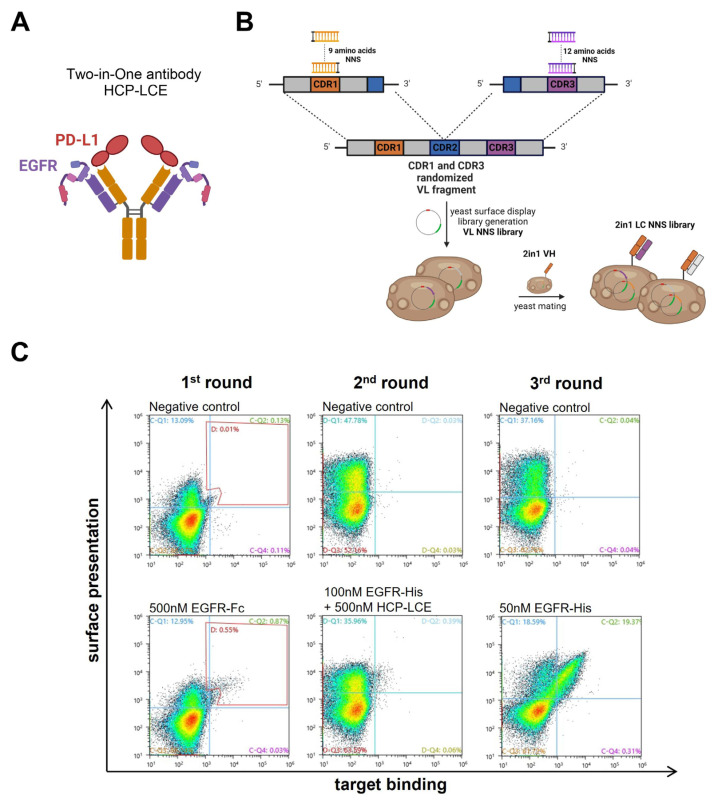
HCP-LCE light chain NNS yeast surface display library generation and sorting. (**A**) Schematic representation of the EGFR and PD-L1 binding Two-in-One antibody HCP-LCE. (**B**) Schematic representation of the cloning procedure for YSD library generation. Single amino acids of LCDR1 and LCDR3 were substituted with a codon encoding for the 20 naturally occurring amino acids. The light chain diversity was combined with the wildtype HCP-LCE heavy chain by yeast mating. Created with BioRender.com (accessed on 11 January 2024) (**C**) Sorting of the diploid HCP-LCE light chain mutant YSD library via FACS. Surface presentation is depicted on the y-axis utilizing the anti-human lambda chain antibody AF647- or PE-labeled, while EGFR-Fc or EGFR-His binding is shown on the x-axis using the anti-human Fc PE antibody or the anti-6xHis AF647 antibody, respectively.

The EGFR-binding module of the Two-in-One antibody HCP-LCE (Figure 1A) was identified via the screening of a chicken-derived yeast surface display library, which was generated by combining the heavy chain of an anti-PD-L1 antibody with an immune anti-EGFR light chain library. Since HCP-LCE targets EGFR and PD-L1 simultaneously with the same Fv region and PD-L1 binding is primarily performed by the heavy chain CDRs, we assume that the light-chain CDRs are mainly involved in EGFR binding [31]. Consequently, in order to mature the EGFR binding affinity of the Two-in-One antibody HCP-LCE, a YSD library was generated by randomizing single amino acids of LCDR1 and LCDR3 (Figure 1B). To minimize the number of mutations, LCDR2 was not modified. Consequently, the YSD library was designed in a way to contain one mutation in any of the nine amino acids of LCDR1 and one mutation in any of the 12 amino acids of LCDR3, resulting in a maximum mutation rate of two mutations per light chain (LC). For this, site-saturation mutagenesis [50] was applied by simultaneously substituting a single LCDR1 and LCDR3 codon with a codon encoding one of the twenty naturally occurring amino acids. To minimize stop codons, degenerated NNS codons were used (Figure 1B). The combination of all possible mutation pairs results in a maximum theoretical library size of 4.3 × 10^4^ light chain mutants on the protein level and 1.1 × 10^5^ mutants on the DNA level. For NNS YSD library cloning, amplified VL fragments were inserted into a pYD_1_-derived vector encoding a human lambda CL by homologous recombination in BJ5464 yeast as described previously [51]. The LC diversity was combined with EBY100 yeast cells encoding the wildtype Two-in-One VH-CH1 fragment by yeast mating (Figure 1B), resulting in adequate oversampling of the calculated light chain diversity.

The diploid Two-in-One LC NNS library was screened for high-affinity EGFR binders by FACS over three consecutive sorting rounds starting with 500 nM EGFR-Fc (Figure 1C). In order to isolate mutants that target EGFR with higher affinity than the wildtype Two-in-One antibody, a competition screen was performed during the second sorting round. For this purpose, the enriched library was stained with 100 nM His-tagged EGFR pre-incubated with 500 nM wildtype Two-in-One antibody (Figure 1C). Double-positive yeast cells are indicative of surface-displayed Fabs that target EGFR with higher affinity than HCP-LCE. After a third round of sorting using 50 nM His-tagged EGFR, six yeast clones were screened individually for EGFR and PD-L1 binding (Figure S1). Since all analyzed clones target both 50 nM EGFR and 250 nM PD-L1, the VL fragment of twelve randomly selected clones of the enriched library after three rounds of sorting was sequenced.

### 3.2. Cloning and Biophysical Characterization of Full-Length Two-in-One Antibody Variants

Of the twelve Two-in-One LC mutants analyzed, the majority contained a wildtype LCDR1 region, suggesting that the light chain CDR1 is not predominantly involved in EGFR binding. Interestingly, all variants showed a mutation at the same position in LCDR3, indicating that this position (amino acid three of LCDR3) has a major impact on EGFR binding. Eleven of the twelve variants had a tyrosine to glutamic acid mutation (Y → E) at position three of LCDR3, and one variant harbored a tyrosine to aspartic acid mutation (Y → D) at this position, which implies the exchange of a polar, neutral amino acid to an acidic amino acid. To investigate the impact of a basic amino acid at position three of LCDR3, a variant carrying a tyrosine to lysine (Y → K) mutation was generated in addition to the variants isolated from the YSD library.

To produce full-length Two-in-One antibody mutants, Expi293F^TM^ cells were co-transfected with the respective VL sequence reformatted into a pTT5-derived vector encoding a lambda CL sequence and a pTT5 vector encoding the Two-in-One heavy chain as previously described [52]. The purification of the HCP-LCE antibody variants, hereafter referred to as LCE-E, LCE-D, and LCE-K depending on the mutation at the LCE light chain, was performed by Protein A affinity chromatography.

SDS-PAGE analysis demonstrated the presence of all expected heavy and light chains under reducing conditions as well as the expected molecular size under non-reducing conditions without degradation products (Figure S2). Thermostability investigated by SYPRO Orange revealed that the melting temperatures of the HCP-LCE variants were between 60.5 °C and 66.5 °C, with the wildtype antibody exhibiting 58.9 °C, indicating no reduction in thermal stability (Table 1).

### 3.3. Characterization of Antigen Binding via Biolayer Interferometry

**Figure 2:**
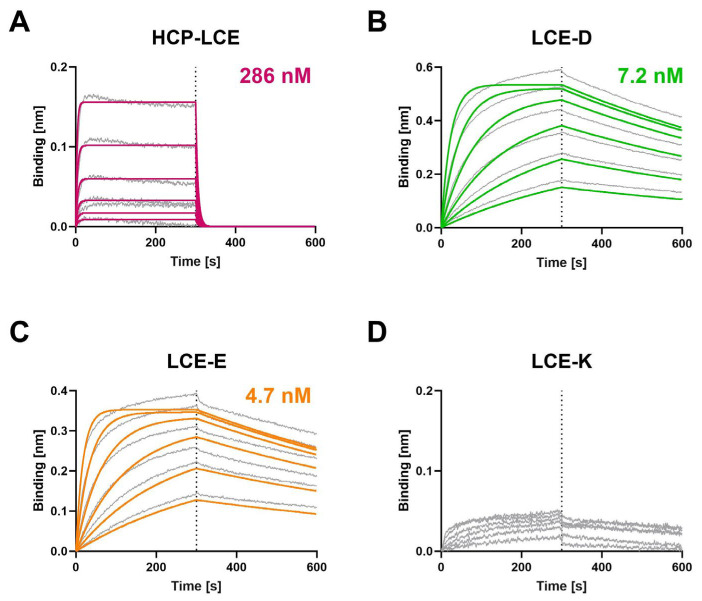
Characterization of the EGFR binding of the HCP-LCE variants by BLI measurements. BLI measurements of (**A**) HCP-LCE, (**B**) LCE-D, (**C**) LCE-E, and (**D**) LCE-K against EGFR. LCE-D and LCE-E target EGFR with a higher affinity than HCP-LCE, whereas the mutant LCE-K is not able to bind EGFR. The fit is depicted by the colored curves.

Biolayer interferometry (BLI) measurements were performed to determine the affinity of the HCP-LCE variants to both EGFR (Figure 2) and PD-L1 (Figure S3). Compared to the HCP-LCE wildtype, the mutants LCE-D and LCE-E exhibit an affinity for EGFR that is increased by a factor of approximately 60. Both LCE-D and LCE-E bind EGFR with a K_D_ value in the single-digit nanomolar range (Figure 2, Table 1). The significant increase in affinity is mainly caused by an improvement in the dissociation rate, which was recorded during the second part of the measurement (300–600 s). The LCE-K mutant does not target EGFR (Figure 2). This indicates that position three in LCDR3 has a strong impact on EGFR binding. A negatively charged amino acid such as aspartic acid (D) or glutamic acid (E) improves binding, whereas a positively charged amino acid like lysine (K) eliminates binding. The antibodies did not show binding to a negative control protein, excluding non-specific binding (Figure S4).

Flow cytometric analysis using yeast cells displaying truncated fragments of the EGFR-ECD confirmed that LCE-D and LCE-E target EGFR at the same domain as described for HCP-LCE [31], suggesting that the LCDR3 mutation does not modify the targeted EGFR epitope (Figure S5). The three antibody variants exclusively target EGFR fragment 1–294 but neither 1–124 nor 1–176, which is why they are mapped to the EGFR dimerization domain II.

All four HCP-LCE variants analyzed target PD-L1 with comparable binding kinetics, indicating that the mutation in LCDR3 does not have a major impact on PD-L1 binding. The PD-L1 binding kinetics are characterized by a fast association rate and a fast dissociation rate, which results in K_D_ values in the double-digit nanomolar range (Figure S3, Table 1). As demonstrated by BLI measurements, the ability to target and block the PD-1/PD-L1 interaction is preserved for variant LCE-E, as the antibody is unable to bind the PD-1/PD-L1 complex (Figure S6). In addition, LCE-E, like the parental antibody HCP-LCE, targets both antigens simultaneously with a single Fab fragment. This was investigated by the immobilization of the one-armed variants of the respective antibodies on BLI biosensors and the subsequent sequential incubation of PD-L1 first and EGFR second (Figure S7).

### 3.4. Real-Time Antigen Binding Measured on Single-Protein and Dual-Protein Surfaces in switchSENSE^®^

**Figure 3:**
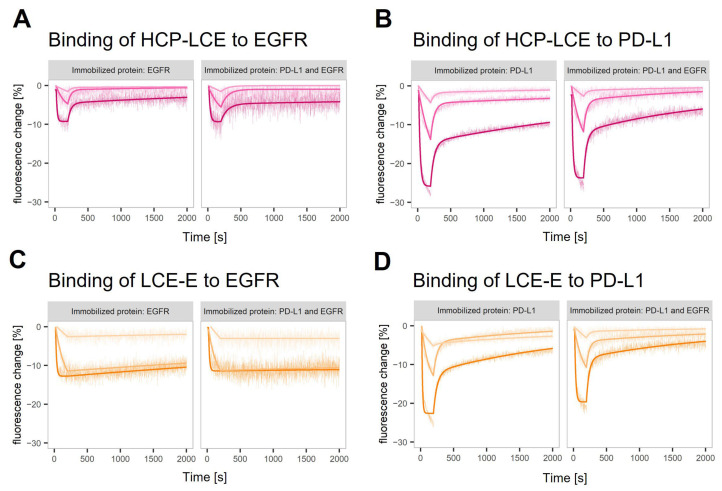
Real-time antigen binding measured on single-protein and dual-protein surfaces. Characterization of (**A**,**C**) EGFR and (**B**,**D**) PD-L1 binding of (**A**,**B**) HCP-LCE and (**C**,**D**) LCE-E on surfaces with EGFR or PD-L1 and on surfaces with both proteins (left and right panel in each plot). The fitting model assumes biphasic dissociation for all observed interactions. The interaction between LCE-E and EGFR shows the lowest contribution of the fast dissociation phase.

The binding kinetics of HCP-LCE and LCE-E to both targets were characterized in a helix^®^ biosensor instrument using switchSENSE^®^ technology. DNA-conjugated targets are hybridized with fluorophore-tagged adapter strands, which hybridize to anchor strands on the chip surface. The binding of the analyte is sensed by the fluorophore, which is sensitive to changes in its local environment. In this case, analyte binding resulted in a quenching effect, visualized as a signal decrease (Figure 3A–D). The technology offers the possibility of using different fluorophores to tag different antigens which can be analyzed in parallel on the same surface in a dual-color experiment. The binding kinetics of the two antibody variants to EGFR and PD-L1, either on surfaces with one immobilized protein or on surfaces where both target proteins were immobilized, were compared. The observed interactions showed biphasic dissociations with a fast and slow dissociation rate (Figure 3). The glutamic acid introduced in LCDR3 (LCE-E variant) reduces the impact of the fast dissociation rate and stabilizes the binding of the antibody to EGFR (Figure 3C). The results obtained on surfaces with one of the target proteins and on surfaces with both target proteins are highly comparable (Table S1). A comparison of the one-armed HCP-LCE and IgG-like HCP-LCE revealed differences in the binding to PD-L1 immobilized on a surface in corresponding density. This indicates that the HCP-LCE kinetic includes the avidity effect caused by bivalent binding to PD-L1. In the given set-up, the presence of EGFR did not stabilize the binding of HCP-LCE or LCE-E to PD-L1.

### 3.5. On-Cell Binding of Affinity-Matured Two-in-One Variants Measured by Flow Cytometry

**Figure 4:**
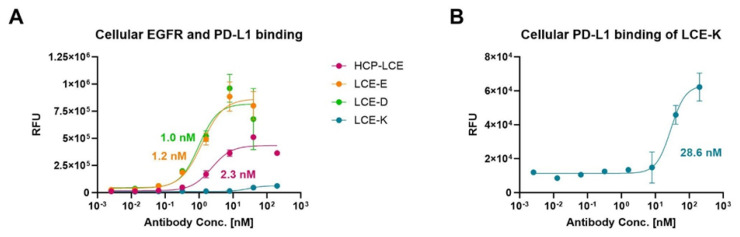
Cellular binding of the HCP-LCE variants on EGFR/PD-L1 double-positive A549 cells. (**A**) Cell titration of HCP-LCE (pink), LCE-E (orange), LCE-D (green), and LCE-K (blue) on A549 cells. (**B**) Cell titration of LCE-K on A549 cells; zoomed-in view of the graph shown in A. A variable slope four-parameter fit was utilized to fit the resulting curves. EC_50_ values: HCP-LCE, 2.3 nM; LCE-E, 1.2 nM; LCE-D, 1.0 nM; LCE-K, 28.6 nM. All measurements were performed in duplicates, and the experiments were repeated at least three times, yielding similar results.

To ensure that the HCP-LCE mutants LCE-E and LCE-D also showed more affine binding to target-positive tumor cells than the wildtype Two-in-One antibody, cellular binding experiments were performed on EGFR and PD-L1 double-positive A549 cells by flow cytometry. The cells were stained with the respective antibody at a concentration ranging from 2.56 pM to 200 nM utilizing a 5-fold dilution series, and binding was verified using an anti-human Fc PE detection antibody. As expected, LCE-E and LCE-D show specific cellular binding, with comparable EC_50_ values of about 1 nM and a similar binding maximum (Figure 4A). The wildtype antibody HCP-LCE targets the tumor cells with lower affinity (EC_50_ = 2.3 nM) and with a significantly reduced maximum binding. The deletion of the EGFR binding property of the mutant LCE-K is also reflected in cellular binding (Figure 4A,B). The overexpression of EGFR on cancer cells usually exceeds that of PD-L1 [51], which is why LCE-K demonstrates a lower EC_50_ value (28.6 nM) and significantly reduced maximum binding. This is also illustrated by the A549 cellular binding of monospecific anti-EGFR and anti-PD-L1 antibodies (Figure S8). The antibodies did not show binding to EGFR and PD-L1 double-negative Jurkat cells, excluding non-specific cellular binding (Figure S9). These data indicate that the amino acid exchange at position three in LCDR3 from tyrosine to glutamic acid (E) or aspartic acid (D) also results in significantly improved tumor cell binding, as expected.

### 3.6. Real-Time Binding Kinetics on Target-Positive Cells Detected with Real-Time Interaction Cytometry

**Figure 5:**
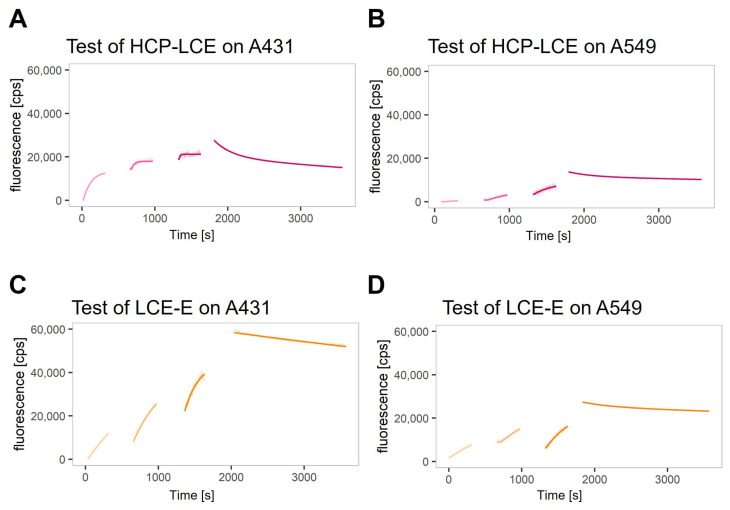
Real-time binding kinetics on EGFR and PD-L1 double-positive A431 and A549 cells. Real-time binding curves of (**A**,**B**) HCP-LCE and (**C**,**D**) LCE-E measured on (**A**,**C**) A431 and (**B**,**D**) A549 via real-time interaction cytometry (RT-IC) in a heliX^cyto^ biosensor. The cells were fixed with PFA and loaded into the five individual cell traps on the chip. Fluorescently labeled analytes were injected in increasing concentrations in three subsequent injection steps. The association of the analytes was visibly reflected by an increase in the fluorescent signal. After the third injection, the system switches to buffer flow to monitor the dissociation of the analytes, which visibly manifests as a decrease in signal. Data points were fitted with a kinetic model that assumes monophasic association and biphasic dissociation.

A real-time interaction cytometry (RT-IC) experiment using heliX^cyto^ biosensor was designed to reveal the impact of the improved EGFR binding kinetics on the binding kinetics of the antibody variant LCE-E and target-positive tumor cells compared to the wildtype HCP-LCE. Binding kinetics were measured using two different EGFR and PD-L1 double-positive tumor cell lines, A431 and A549. On A431 cells, expression levels for EGFR and PD-L1 are higher compared to A549 cells, with EGFR expression being higher than the expression of PD-L1 on both cell lines [51]. The cells were loaded into the cell traps on the chip of the heliX^cyto^ biosensor, and fluorescently labeled antibody variants were injected in three injection steps at increasing antibody concentrations. The association of the antibodies was visibly reflected by an increase in the fluorescence signal; the dissociation was visibly reflected by a decrease in the signal after switching to buffer flow. The binding of both antibodies (HCP-LCE and LCE-E) was detectable on A431 cells and A549 cells (Figure 5A–D).

**Figure 6:**
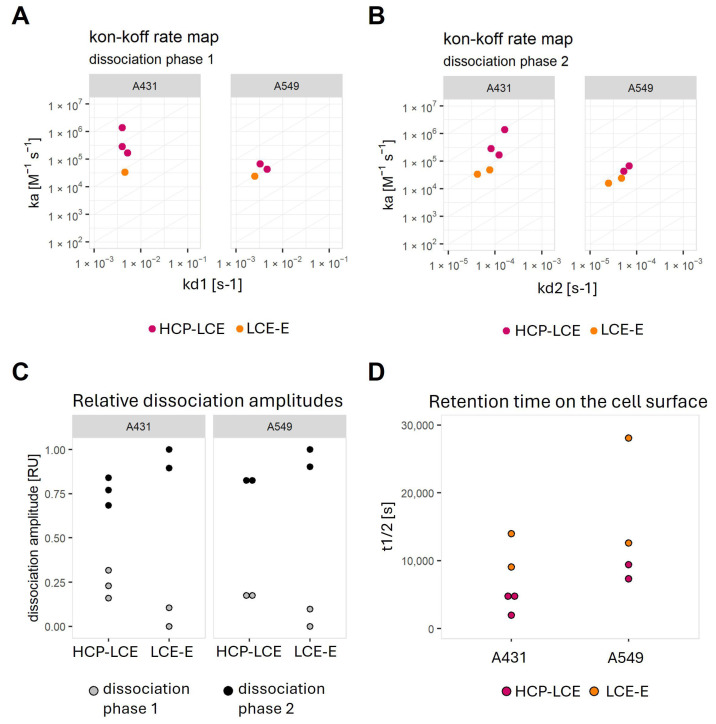
Graphical representation of calculated kinetic values after fitting the binding curves with a kinetic model that assumes the monophasic association and biphasic dissociation, as well as the complete dissociation, of the analyte. On–off-rate maps plotting (**A**) the association rate versus dissociation rate 1 or (**B**) versus dissociation rate 2. (**C**) Plot demonstrating the relative contribution of the faster dissociation rate (dissociation phase 1) and the slower dissociation rate (dissociation phase 2). (**D**) Plot of half-life values calculated from the fit model for each interaction.

In most cases, the dissociation rate was biphasic and dominated by the slower dissociation rate (Figure 6). The K_D_ values calculated with the slower dissociation rate (KD2) are in the range of 0.1–0.7 nM for HCP-LCE on A431 cells and 1–2 nM for all other measured interactions (Table S2). The lower KD2 values determined for HCP-LCE on A431 were caused by a faster association rate (Figure 6A,B).

On both cell lines, LCE-E shows a slower second dissociation rate (kd2) (Figure 6C,D) and a weaker contribution of the second dissociation phase to the total dissociation amplitude (Figure 6C). This results in an increase in the length of time for which the affinity-matured antibody is retained on the cell surface (Figure 6D).

### 3.7. Characterization of Antigen Binding via AlphaFold-Based Modeling

**Figure 7:**
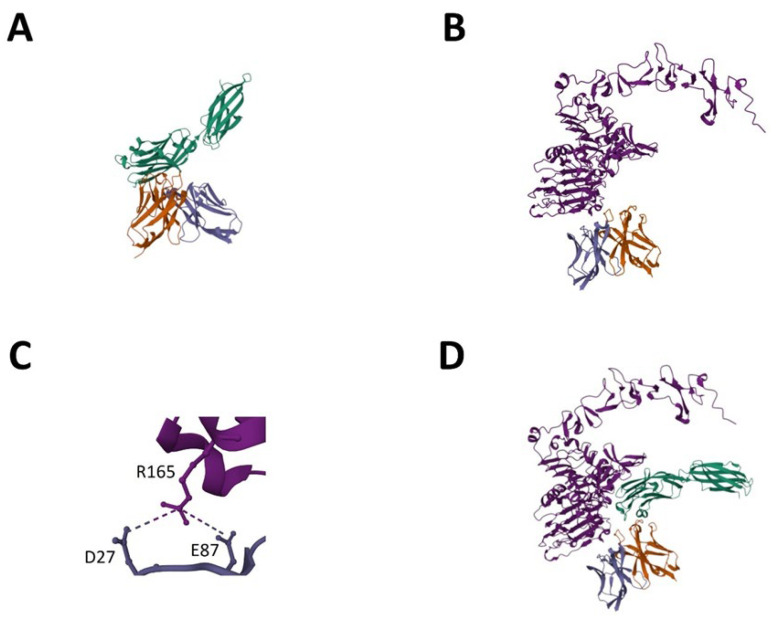
AlphaFold-based model for EGFR and PD-L1 binding of LCE-E. (**A**) PD-L1-ECD (green) binding of LCE-E. (**B**) EGFR-ECD (light purple) binding of LCE-E. (**C**) Salt bridge between the glutamic acid at LCDR3 of LCE-E (E87) and an arginine at EGFR-ECD position 165 (R165). (**D**) Tetrameric protein complex of PD-L1 docked to the EGFR:VH:VL complex. The VH fragment is shown in orange, and the VL fragment is shown in dark purple. Created with AlphaFold Multimer.

In order to investigate the protein–protein interaction of the LCE-E mutant and the wildtype HCP-LCE with its antigens EGFR and PD-L1 in more detail, AlphaFold Multimer was used [38,39]. As experimentally predicted for HCP-LCE [31], PD-L1 binding occurs mainly via the heavy chain CDRs (Figure 7A), whereas heavy and light chain CDRs are involved in EGFR binding (Figure 7B). The AlphaFold-based model predicts that HCDR3 and LCDR3 mainly target the EGFR epitope. This is consistent with the experimentally obtained data showing an impact of LCDR3 on EGFR binding. Remarkably, the predicted EGFR epitope, EGFR dimerization domain II, is consistent with the epitope experimentally determined by YSD-based epitope mapping. To predict the affinity of the protein–protein complexes, the PRODIGY web server was utilized [46]. For HCP-LCE binding to EGFR, a K_D_ value of 110 nM was predicted, and for LCE-E binding to EGFR, a K_D_ value of 6.2 nM was predicted (Table 1). Thus, the affinity prediction also implies a significant increase in affinity as a result of the amino acid exchange at LCDR3 position three. Structural insights indicate that the increased affinity is caused by the formation of a salt bridge between the glutamic acid at LCDR3 position three (E87) and an arginine at EGFR position 165 (R165) (Figure 7C). The binding of the parental antibody HCP-LCE involves the formation of a salt bridge between EGFR R165 and D27, which is also observed for the interaction of EGFR and LCE-E.

To demonstrate that the Two-in-One VH:VL dimer can target EGFR and PD-L1 simultaneously, a protein docking method was applied [48,53]. PD-L1 was docked to the EGFR:VH:VL complex, resulting in a tetrameric protein complex in which both antigens can bind simultaneously without steric hindrance (Figure 7D). PAE and pLDDT plots generated using AlphaFold Multimer are shown in Figure S10. The computer-based data are very consistent with the experimentally determined data.

## 4. Discussion

Affinity maturation is the process in which antibodies obtain increased affinity and functionality. It is the result of the somatic hypermutation of immunoglobulin genes in B cells, coupled with selection for antigen binding, a process that occurs over weeks after an acute infection or vaccination [54]. The resulting antibodies can be highly mutated compared to their germline-encoded counterparts, with the affinity for antigens being significantly increased compared to the corresponding naïve B cell receptors [55,56]. This makes affinity maturation a key technique in protein engineering to improve affinity and binding interactions in vitro in order to optimize the therapeutic potential of antibodies [57]. Adler et al. observed that in vivo affinity-matured antibodies predominantly accumulate mutations within the CDR3 regions, with a total of 6–7 amino acid changes in the heavy and light chain [58]. However, during in vitro affinity maturation, libraries with a mutation rate of 1–2 are sufficient for isolating high-affinity binders [59].

In this study, we generated affinity-matured variants of a chicken-derived EGFR×PD-L1 Two-in-One antibody that are enhanced in terms of their EGFR binding properties. To this end, site-saturation mutagenesis was performed by simultaneously substituting a single LCDR1 and LCDR3 codon with degenerated NNS codons. FACS screening of the randomized YSD library resulted in the isolation of a Two-in-One variant (LCE-E) that exhibited a 60-fold improvement in EGFR binding affinity due to the exchange of a single amino acid at LCDR3 position three (Y → E). An AlphaFold-based model showed that the improved EGFR affinity is likely caused by the formation of a salt bridge between the positively charged glutamic acid at LCDR3 position three and a negatively charged arginine at EGFR position 165. A salt bridge is the strongest type of non-covalent interaction in nature, and salt bridges are known to be involved in protein–protein interactions and molecular recognition. A salt bridge combines an electrostatic attraction between oppositely charged chemical groups and a hydrogen bond, which is why its strength exceeds that of a simple hydrogen bond [60,61,62]. The LCE-D variant, which contains the amino acid aspartic acid at LCDR3 position three (Y → D), also shows significantly improved EGFR binding comparable to LCE-E. This indicates that the positive charge at this position has a major impact on EGFR binding. A negative charge at LCDR3 position three, as found in the LCE-K mutant (Y → K), eliminates EGFR binding, further supporting this hypothesis.

The improved EGFR binding affinity of the Two-in-One variant LCE-E was confirmed using various methods. The BLI measurements, as well as real-time antigen binding measurements on mixed surfaces and cellular binding experiments on tumor cells, showed significantly improved EGFR binding compared to the wildtype HCP-LCE. In particular, the dissociation rate of LCE-E was reduced, resulting in a stabilized binding and a longer retention time on the cell surface.

However, a higher affinity does not guarantee an improved clinical efficacy. One example for this is a variant of palivizumab with 44-fold-improved potency which only demonstrated a modest improvement in efficacy in subsequent in vivo studies and a poor pharmacokinetic profile due to non-specific binding [23].

HCP-LCE is a chimeric antibody consisting of chicken-derived VH and VL domains grafted onto a human IgG1 scaffold [31]. Unlike in mammals, the rearrangement of the variable (V), diverse (D), and connecting (J) gene segments in chickens occurs simultaneously in the heavy and light chains [63,64]. In avian species, however, there is only a small selection of immunoglobulin genes during V(D)J gene rearrangement. Therefore, somatic gene conversion and somatic hypermutation are utilized to further diversify the variable gene [65,66]. The generation of the heavy and light chain repertoire of a chicken thus differs considerably from that of rodents such as mice or rats, which are commonly used for antigen immunization. This could be one reason for the success of affinity maturation based on the targeted mutation of the variable CDR regions of the chicken-derived antibody.

One advantage of antibody generation after animal immunization is that the antibodies have already undergone in vivo affinity maturation [8]. Since two chickens were immunized for the generation of HCP-LCE [31] and the heavy chain of an anti-PD-L1 antibody was paired with an anti-EGFR light chain diversity, the heavy and light chain of the Two-in-One antibody were never present in the same chicken. Therefore, no in vivo affinity maturation occurred for this heavy and light chain combination, demonstrating the potential for in vitro affinity optimization.

HCP-LCE simultaneously targets EGFR and PD-L1 at the same Fv fragment, inhibiting EGFR signaling by binding to dimerization domain II and blocking the PD-1/PD-L1 interaction [31]. The advantageous combination of the antigens EGFR and PD-L1 has also been described in other studies. Koopmans et al. constructed a bispecific PD-L1×EGFR antibody to direct PD-L1 blockade to EGFR-expressing cancer cells [67]. By fusing a dual-targeting tandem trimmer body with the human IgG1 hinge and Fc region, Rubio-Pérez et al. generated a symmetric bispecific PD-L1×EGFR antibody that combines an immune checkpoint blockade with a direct action on cancer cells [68]. It is assumed that this target combination improves the efficacy of current immunotherapies.

Overall, we have presented a straightforward method for the affinity maturation of chicken-derived Two-in-One antibodies, optimizing the affinity of only one of the two targets addressed simultaneously with a single Fab fragment. The mutation of individual amino acids of the LCDR3 region followed by YSD library generation and FACS screening resulted in the isolation of a variant that targets EGFR with a 60-fold-increased affinity. BLI measurements demonstrated that the increase in affinity is mainly caused by an improvement in the dissociation rate. LCE-E and LCE-D demonstrated specific cellular binding properties on EGFR/PD-L1 double-positive tumor cells with higher affinity and improved maximum binding compared to the wildtype HCP-LCE. AlphaFold-based models predict that the glutamic acid in LCDR3 forms a salt bridge to EGFR, which causes the increase in affinity. The exchange of glutamic acid to lysine (LCE-K) completely deletes the EGFR binding property. To our knowledge, this represents the first affinity-matured Two-in-One antibody with optimized binding for one of its two targets.

## Figures and Tables

**Table 1:** Biophysical properties of HCP-LCE, LCE-E, LCE-D, and LCE-K, including BLI measured affinity and kinetic binding rates, flow cytometry-measured EC_50_ values, PRODIGY web server predicted affinity values, and melting temperatures.

Antibody	K_D_ [nM]		k_on_ [M^−1^ s^−1^]		K_dis_ [s^−1^]		R^2^		EC_50_ A549 [nM]	Predicted K_D_ [nM]	T_M_ [°C]
	EGFR	PD-L1	EGFR	PD-L1	EGFR	PD-L1	EGFR	PD-L1		EGFR	
HCP-LCE	286	70.7	6.13 × 10^5^	1.42 × 10^5^	1.75 × 10^−1^	1.01 × 10^−2^	0.85	0.89	2.3	110	58.9
LCE-E	4.7	52.2	2.25 × 10^5^	1.54 × 10^5^	1.06 × 10^−3^	8.02 × 10^−3^	0.96	0.84	1.2	6.2	60.5
LCE-D	7.2	25.6	1.66 × 10^5^	5.63 × 10^5^	1.19 × 10^−3^	1.44 × 10^−2^	0.97	0.96	1.0	not determined	61.0
LCE-K	-	23.8	-	5.30 × 10^5^	-	1.26 × 10^−2^	-	0.98	28.6	not determined	66.5

## Data Availability

The data pertaining to this study are available in the manuscript.

## Supplementary Materials

The following supporting information can be downloaded at: https://www.mdpi.com/article/10.3390/antib13020036/s1 Figure S1: Flow cytometric analysis of six isolated yeast single clones after three consecutive rounds of FACS screening; Figure S2: SDS-PAGE analysis of the wildtype Two-in-One antibody HCP-LCE and mutants LCE-E, LCE-D, and LCE-K under reducing (left) and non-reducing conditions (right); Figure S3: Characterization of PD-L1 binding of the HCP-LCE variants by BLI measurements; Figure S4: Characterization of (A) HCP-LCE, (B) LCE-D, (C) LCE-E, and (D) LCE-K binding to a negative control protein by BLI measurements; Figure S5: YSD-based EGFR epitope mapping; Figure S6: BLI-assisted PD-1 competition assay; Figure S7: BLI-assisted simultaneous binding assay; Figure S8: Cellular binding of a monospecific anti-EGFR (grey) and anti-PD-L1 antibody (blue) compared to LCE-E (orange) on EGFR/PD-L1 double-positive A549 cells; Figure S9: Cellular binding of the HCP-LCE variants on EGFR/PD-L1 double-negative Jurkat cells; Figure S10: pLDDT and PAE plots generated by AlphaFold Multimer; Table S1: Kinetic values calculated from switchSENSE^®^ measurements; Table S2: Kinetic values calculated from RT-IC measurements.
