# Supplementary material for: Balancing the Affinity and Tumor Cell Binding of a Two-in-One Antibody Simultaneously Targeting EGFR and PD-L1
Source: Antibodies (Basel). 2024 May 2;13(2):36. doi: 10.3390/antib13020036 (PMC11130809; doi:10.3390/antib13020036)
Supplement: Supplementary file 1 [file antibodies-13-00036-s001.zip › antibodies-2889773-supplementary.pdf]

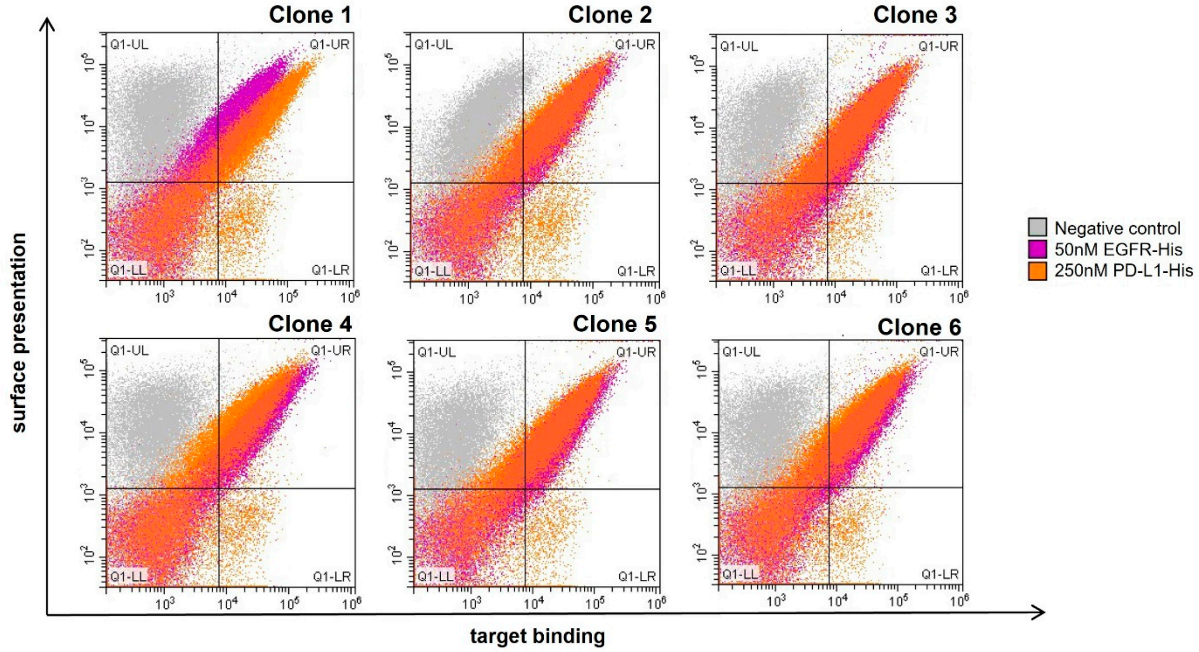

Figure S1. Flow cytometric analysis of six isolated yeast single clones after three consecutive rounds of FACS screening. Surface presentation is depicted on the y-axis utilizing the anti-human-Lambda PE-conjugated F(ab´)2 antibody, while EGFR-His$_6$ (purple) and PD-L1-His$_6$ (orange) binding is shown on the x-axis using the anti-6xHis AF647 antibody.

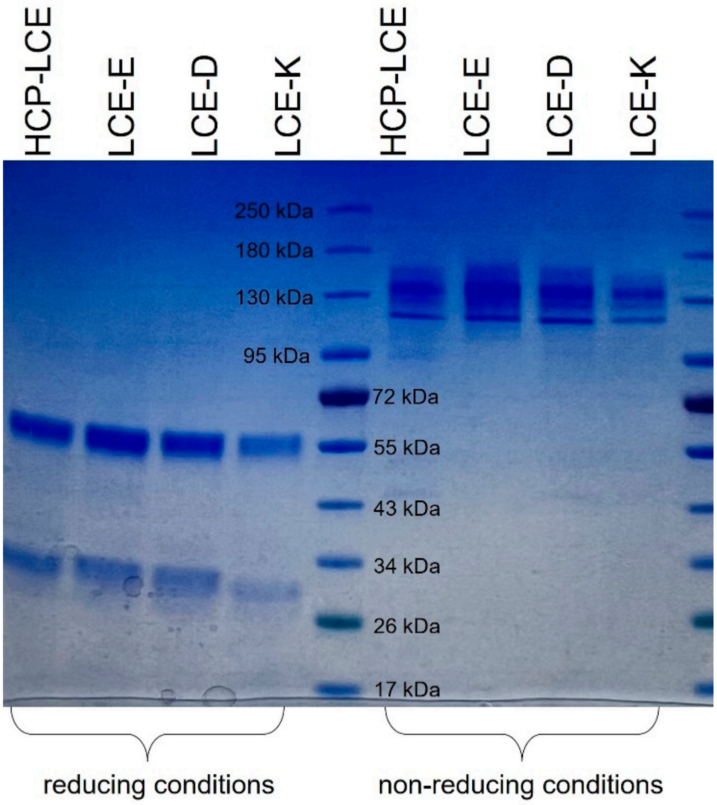

Figure S2. SDS-PAGE analysis of the wildtype Two-in-One antibody HCP-LCE and mutants LCE-E, LCE-D and LCE-K under reducing (left) and non-reducing conditions (right). As protein standard, Color Prestained Protein standard (New England Biolabs) was used.

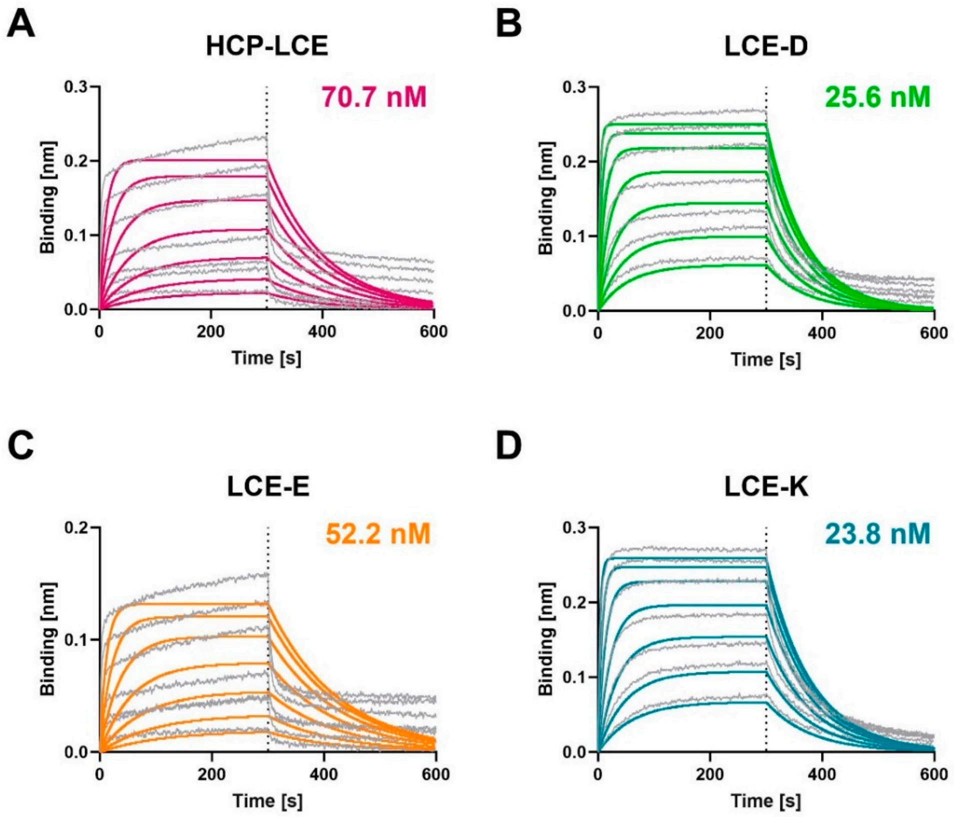

Figure S3. Characterization of PD-L1 binding of the HCP-LCE variants by BLI-measurements. BLI-measurements of (A) HCP-LCE, (B) LCE-D, (C) LCE-E and (D) LCE-K against PD-L1. The fit is depicted by the colored curves.

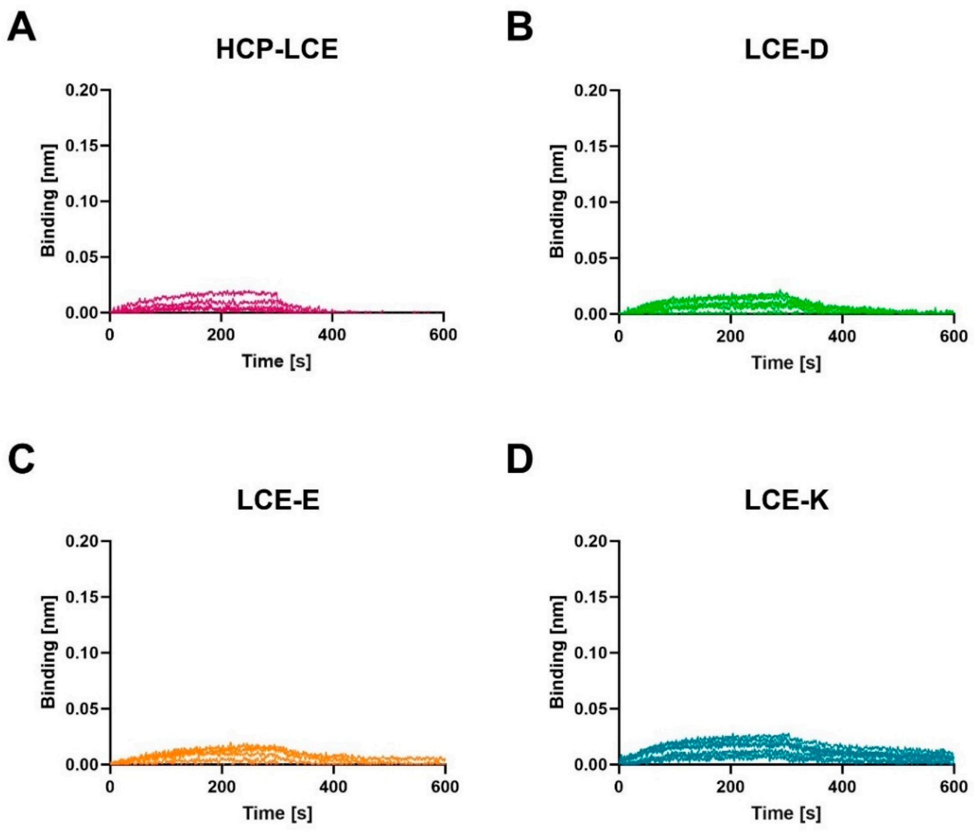

Figure S4. Characterization of (A) HCP-LCE, (B) LCE-D, (C) LCE-E and (D) LCE-K binding to a negative control protein by BLI-measurements.

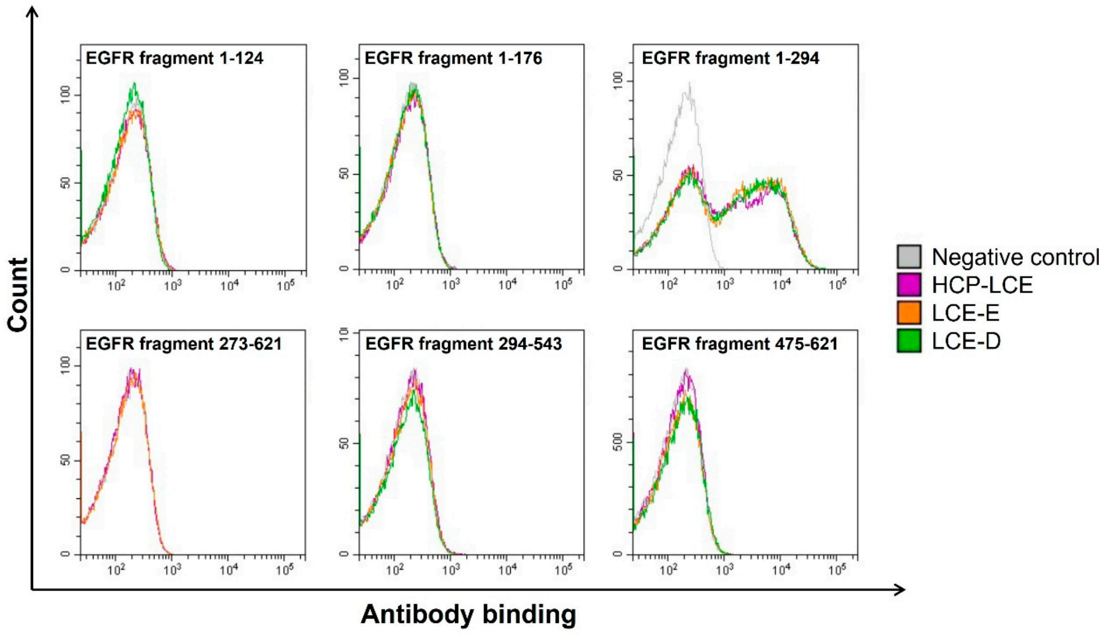

Figure S5. YSD-based EGFR epitope mapping. Binding of HCP-LCE (pink), LCE-E (orange) and LCE-D (green) to yeast cells expressing different truncated EGFR fragments was detected using the anti-human Fc PE-labelled antibody. Measurements

without antibody (grey) served as negative control. All antibodies target EGFR fragment 1-294.

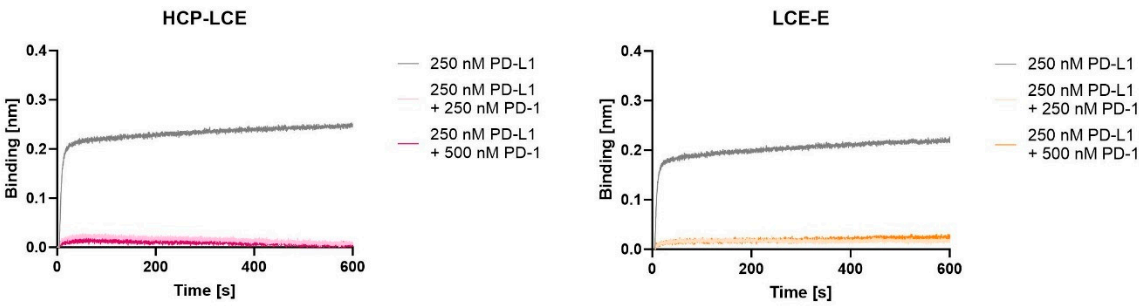

Figure S6. BLI-assisted PD-1 competition assay. HCP-LCE and LCE-E were loaded onto FAB2G biosensors and subsequently associated to PD-L1 pre-incubated with varying PD-1 concentrations. The antibodies do not target the PD-1/PD-L1 complex.

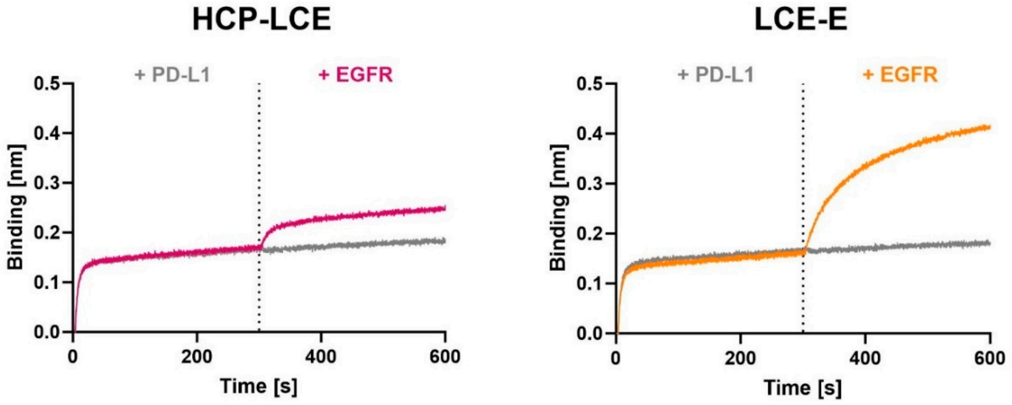

Figure S7. BLI-assisted simultaneous binding assay. One-armed variants of the antibodies HCP-LCE and LCE-E were loaded onto AHC biosensors and antigens were added stepwise, revealing simultaneous PD-L1 and EGFR binding.

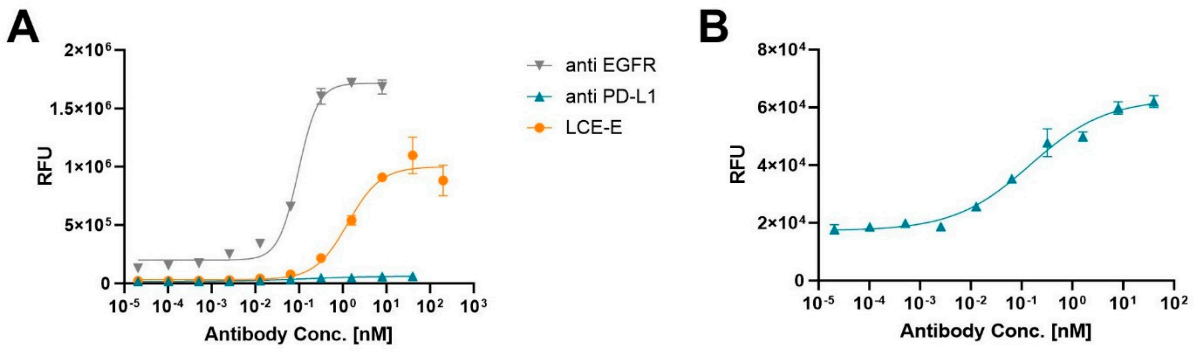

Figure S8. Cellular binding of a monospecific anti-EGFR (grey) and anti-PD-L1 antibody (blue) compared to LCE-E (orange) on EGFR/PD-L1 double positive A549 cells. B is a zoomed-in view of the graph shown in A.

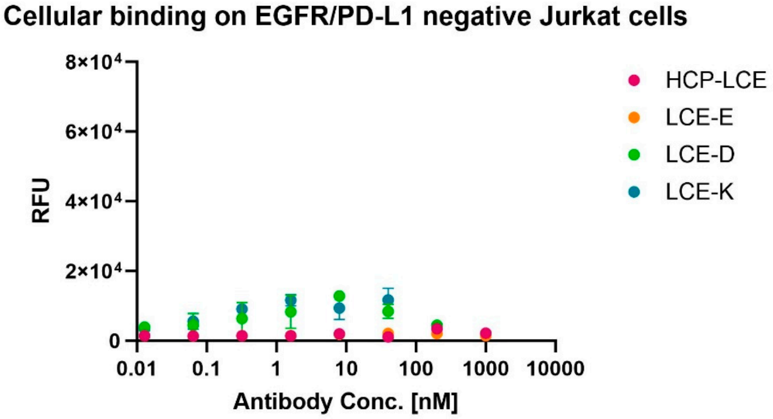

Figure S9. Cellular binding of the HCP-LCE variants on EGFR/PD-L1 double negative Jurkat cells. Cell titration of HCP-LCE (pink), LCE-E (orange), LCE-D (green) and LCE-K (blue) on Jurkat cells.

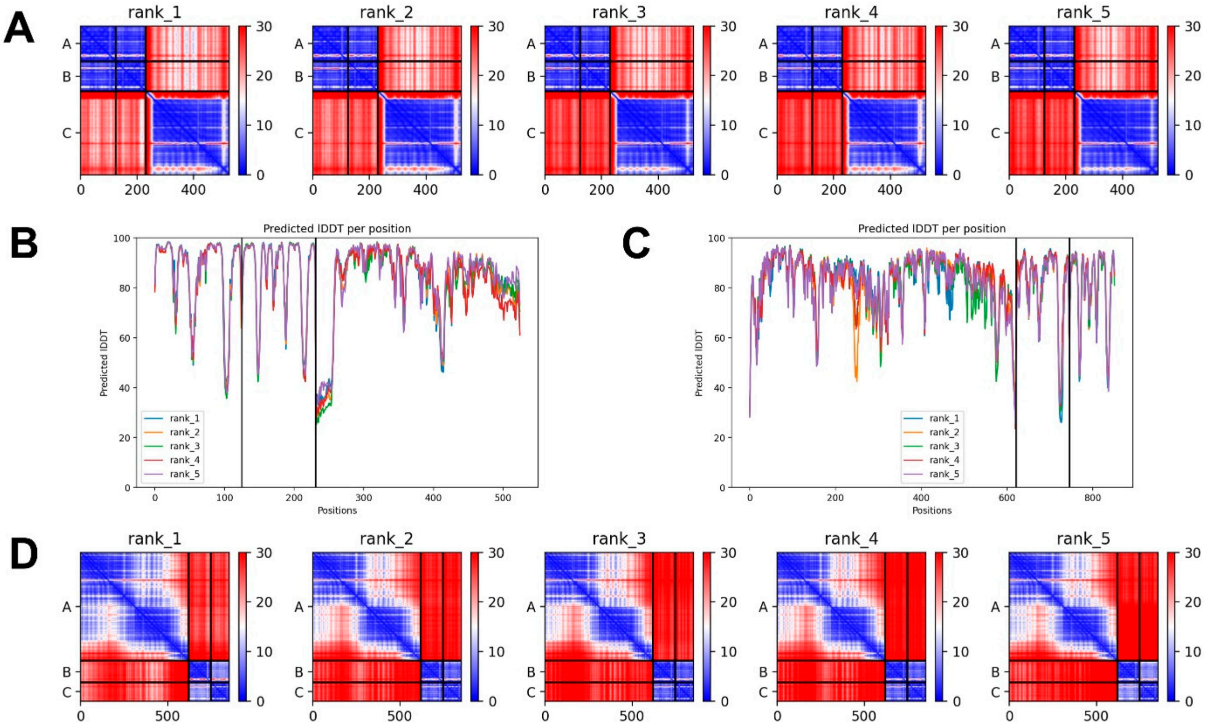

Figure S10. pLDDT and PAE plots generated by AlphaFold Multimer. (A) PAE plots of LCE-E and EGFR modelling, (B) pLLDT plot of LCE-E and EGFR modelling, (C) pLLDT plot of HCP-LCE and EGFR modelling, (D) PAE plots of HCP-LCE and EGFR modelling.

Table S1. Kinetic values calculated from switchSENSE® measurements.

analyte	surface	monitored interaction	ka [$M^{-1}s^{-1}$]	Kd1 [s^{-1}]	Kd2 [s^{-1}]	KD1 [M]	KD2 [M]	relative dissociation amplitude 1	relative dissociation amplitude 2	$t_{1/2}$ [s]
HCP-LCE	PD-L1	PD-L1	4.42E+06	1.87E-02	2.66E-04	4.23E-09	6.02E-11	0.603	0.397	90
	EGFR and PD-L1	EGFR	5.08E+06	1.04E-02	6.88E-05	2.05E-09	1.36E-11	0.795	0.205	95
		PD-L1	5.29E+06	2.17E-02	5.34E-04	4.10E-09	1.01E-10	0.607	0.393	74
	EGFR	EGFR	7.28E+06	2.25E-02	3.19E-04	3.09E-09	4.39E-11	0.690	0.310	56
LCE-E	PD-L1	PD-L1	9.27E+06	1.79E-02	4.71E-04	1.94E-09	5.08E-11	0.436	0.564	269
	EGFR and PD-L1	EGFR	7.39E+06	NA	2.11E-04	NA	2.86E-11	NA	NA	3283
		PD-L1	6.15E+06	2.31E-02	4.85E-04	3.76E-09	7.88E-11	0.626	0.374	35
	EGFR	EGFR	6.78E+06	1.33E-04	1.06E-04	1.96E-11	1.56E-11	0.392	0.608	5982

Table S2. Kinetic values calculated from RT-IC measurements.

analyte	cell line	ka [$M^{-1}s^{-1}$]	kd1 [s^{-1}]	kd2 [s^{-1}]	KD1 [M]	KD2 [M]	relative dissociation amplitude 1	relative dissociation amplitude 2	$t_{1/2}$ [s]
HCP-LCE	A431	1.37E+06	3.97E-03	1.59E-04	2.90E-09	1.16E-10	0.316	0.684	1966
		2.81E+05	3.97E-03	8.21E-05	1.41E-08	2.92E-10	0.229	0.771	5268
		1.66E+05	5.18E-03	1.21E-04	3.12E-08	7.30E-10	0.159	0.841	4291
	A549	6.70E+04	3.28E-03	6.89E-05	4.89E-08	1.03E-09	0.172	0.828	7312
		4.31E+04	4.65E-03	5.30E-05	1.08E-07	1.23E-09	0.177	0.823	9389
LCE-E	A431	3.41E+04	4.52E-03	4.17E-05	1.33E-07	1.23E-09	0.105	0.895	13960
		4.89E+04	NA	7.67E-05	NA	1.57E-09	0.000	1.000	9035
	A549	1.61E+04	NA	2.47E-05	NA	1.53E-09	0.000	1.000	28056
		2.43E+04	2.50E-03	4.71E-05	1.03E-07	1.93E-09	0.096	0.904	12573
